# Ultrasound assessment of the brachial plexus nerve root cross-sectional areas in asymptomatic patients with type 2 diabetes

**DOI:** 10.1097/MD.0000000000036806

**Published:** 2023-12-29

**Authors:** Mamdouh Ali Kotb, Mohamed A. Bedewi, Daifallah Mohamed Almalki, Ali Abdullah AlAseeri, Kholoud J. Sandougah, Steven B. Soliman, Nasser M. Aldossary, Wael Hamed Aboulela

**Affiliations:** a Department of Internal Medicine, Prince Sattam Bin Abdulaziz University, College of Medicine, Al-Kharj, Kingdom of Saudi Arabia; b Neurology Department, Faculty of Medicine, Minia University, Minia, Egypt; c Department of Medicine, College of Medicine, Al Imam Mohammed Ibn Saud Islamic University, Riyadh, Saudi Arabia; d Division of Musculoskeletal Radiology, Department of Radiology, University of Michigan, Ann Arbor, MI; e Neurosurgery Department, Faculty of Medicine, Minia University, Minia, Egypt.

**Keywords:** brachial plexus, cross-sectional area, diabetes mellitus, ultrasound

## Abstract

Type 2 diabetes mellitus (T2D) is one of the most common metabolic diseases and is often associated with cervical radiculoplexus neuropathies. Magnetic resonance imaging is the modality of choice for evaluating the brachial plexus, however, the use of ultrasound for its evaluation has increased and has been shown to be an additional reliable method. We aimed to compare the cross-sectional areas of the C5, C6, and C7 nerve roots of the brachial plexus, at the interscalene groove, in asymptomatic patients with T2D to that of an asymptomatic control cohort without T2D. A total of 25 asymptomatic patients with T2D were recruited from outpatient clinics. A total of 18 asymptomatic subjects without T2D were also recruited from hospital staff volunteers to form the control cohort. High-resolution ultrasound imaging of the bilateral C5, C6, and C7 nerve roots of the brachial plexus was performed in the short axis, at the level of the interscalene grooves. The nerve root cross-sectional areas were recorded and compared. In the patients with T2D, HbA1c and fasting blood glucose (FBG) levels were obtained as well as the duration of T2D in years and correlated with cross-sectional areas. The cross-sectional areas of C6 and C7 were significantly smaller in the T2D cohort. Additionally, HbA1c, and FBG levels as well as the duration of T2D were negatively correlated with the C5, C6, and C7 cross-sectional areas. Our study demonstrated smaller brachial plexus nerve root cross-sectional areas in asymptomatic patients with T2D which negatively correlated with HbA1c, and FBG levels as well as the duration of T2D.

## 1. Introduction

Type 2 diabetes mellitus (T2D) is one of the most common metabolic diseases. It can be associated with sensory or sensorimotor polyneuropathy, autonomic neuropathy, polyradiculoneuropathies, cranial neuropathy, and other mononeuropathies.^[1]^ T2D is widely accepted to be associated with lumbosacral radiculoplexus neuropathy, and can also be associated with cervical radiculoplexus neuropathies.^[2]^ The anterior roots of C5 to T1, which are located between the anterior and medial scalene muscles, form the brachial plexus that serves as the sensorimotor innervation of the upper extremity. The upper trunk of the brachial plexus is formed from the C5 and C6 nerve roots, the middle trunk from C7, and the lower trunk from C8 and T1.^[3]^ Patients with diabetic cervical radiculoplexus neuropathies (DCRPN) can present with acute onset of upper extremity pain, weakness, and sensory symptoms. The onset tends to be predominantly unilateral with subsequent spread to the contralateral side.^[2]^ Patients frequently display nonspecific inflammatory markers from a widespread immune response with a predominantly axonal process on electroneurophysiological studies. Magnetic resonance imaging shows variable abnormalities including abnormally hyperintense T2 signal in the edematous nerve, diffuse nerve enlargement, fascicular enlargement, contrast enhancement, muscle denervation edema in the subacute stage, and fatty atrophy of the muscle in the chronic stage.^[2]^

High-resolution ultrasound (US) has been used extensively for years in the evaluation of peripheral and cranial nerves.^[4–8]^ Recently, US cross-sectional area (CSA) reference values of the cervical 5 (C5), cervical 6 (C6), and cervical 7 (C7) brachial plexus roots at the interscalene groove were reported in healthy subjects.^[9]^

We aimed to evaluate the CSA of the C5, C6, and C7 nerve roots at the interscalene groove in asymptomatic patients with T2D as compared to a control cohort.

## 2. Subjects and methods

### 2.1. Selection of study cohorts

Asymptomatic patients with T2D were recruited from outpatient internal medicine and family medicine clinics of Prince Sattam Bin Abdulaziz University hospital. Patients with DCRPN or any other medical condition that may cause radiculoplexus neuropathies were excluded. This resulted in a cohort of 25 patients with T2D.

The control cohort was comprised of healthy asymptomatic volunteers from our hospital staff who had no history of diabetes, impaired glucose tolerance/prediabetes, a neurodegenerative disease, or any other relevant chronic condition. At total of 18 patients comprised this control cohort.

Demographic information about age and gender as well as height, weight, and body mass index were recorded from all participants. Medical history was taken from the patients including onset, course, and duration of T2D as well as medical treatments. HbA1c and fasting blood glucose (FBG) levels were also obtained.

This study was performed in accordance with the ethical standards of our institutional research committee and with the 1964 Declaration of Helsinki and its later amendments or comparable ethical standards. Institutional review board approval was obtained for this prospective study, and informed consent was obtained from all participants prior to study enrollment. Our study complied with the Health Insurance Portability and Accountability Act.

### 2.2. Brachial plexus nerve roots US technique

**Figure 1.**
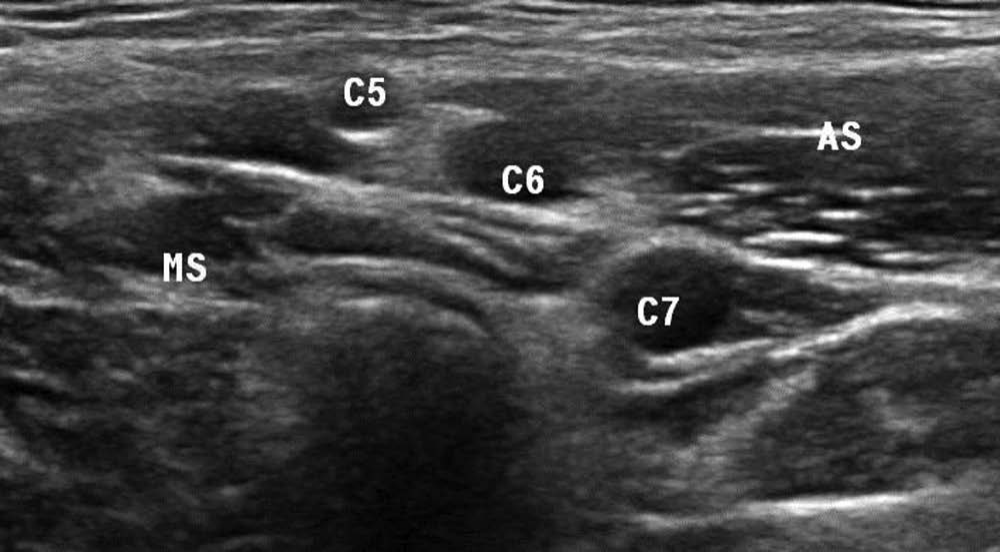
Short-axis sonographic image of the C5-C7 brachial plexus nerve roots, in the interscalene groove, in a patient with type 2 diabetes AS = Anterior scalene muscle, MS = middle scalene muscle.

**Figure 2.**
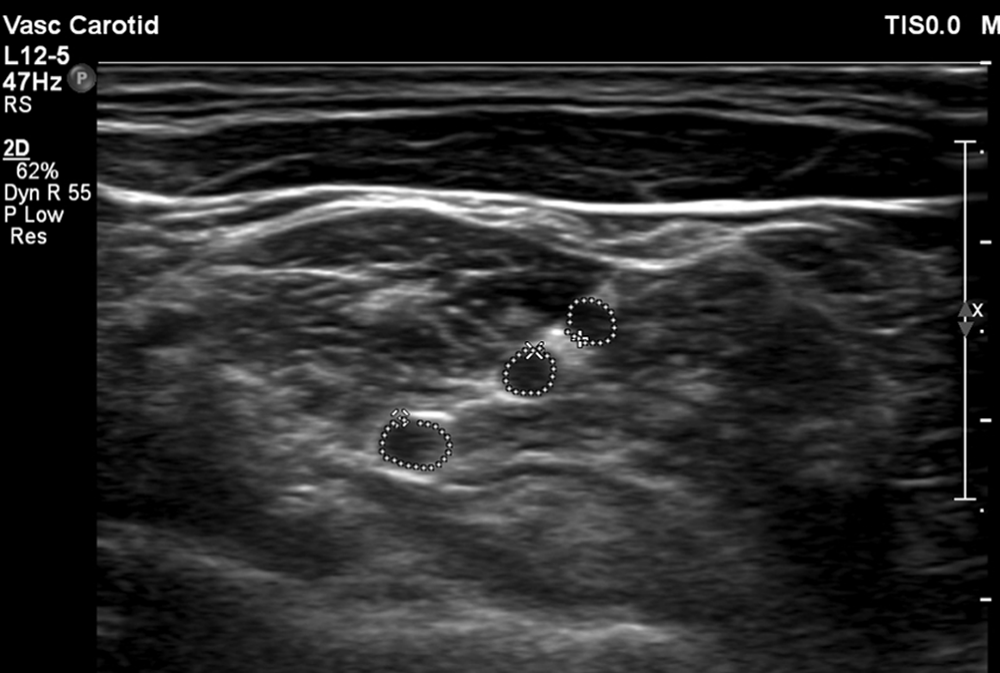
Short-axis sonographic image of the C5-C7 nerve root cross-sectional areas obtained with tracer measurements.

High-resolution ultrasound imaging of the bilateral C5, C6, and C7 nerve roots of the brachial plexus was performed in the short axis, at the level of the interscalene grooves. All subjects were placed in the supine position and the US transducer was positioned superior and lateral to each thyroid lobe. The anterior and middle scalene muscles were identified and the brachial plexus nerve roots were identified as rounded/oval hypoechoic structures (Figs. 1 and 2). The C5 and C6 nerve roots were seen in the short axis between the anterior and posterior tubercles of the transverse process, and the C7 nerve root was seen with the small anterior tubercle and normal posterior tubercle. Each subject was scanned 3 times with transducer removal from the skin between each image and measurements. The C5, C6, and C7 nerve root CSAs were obtained by trace measurements. All images and measurements were obtained by one experienced radiologist (M.B, 15 years of experience in musculoskeletal US) utilizing an L18–5MHz linear transducer (Epic 7 version1.5, Ultrasound system: Philips, Bothell, WA). An experienced neurologist (M.K) reviewed the images for all the studies.

### 2.3. Statistical analysis

Statistical analyses were performed using the IBM Statistical Package for the Social Sciences (SPSS) for Windows, version 26 (IBM Corp., Armonk, NY). The normal distribution of variables was tested using the Kolmogorov–Smirnov test. Descriptive statistics were used for mean, stander deviation and percentage of variables. Paired sample *t* test was used to compare between the right and left sides. Independent sample *t* test was used for parametric data. The relationship between the bilateral C5, C6, andC7 mean nerve root CSAs and the age, weight, height, and body mass index of both cohorts as well as the FBG, HbA1c, and duration of T2D in the T2D cohort was performed using Pearson correlation coefficient. Statistical significance was defined as a *P* < .05.

## 3. Results

The present study included 25 patients with T2D (49 C5, C6, and C7 nerve roots; 64% were men) and 18 asymptomatic control subjects (36 C5, C6, and C7 nerve roots; 61.1% were men). There was no significant difference (*P* = .099) between the mean age of the T2D cohort (58.5 ± 10.6 years) and the control cohort (51.6 ± 14.6 years). Height, weight, and body mass index were also not significantly different (*P* = .453, 0.181, and 0.285, respectively) between the 2 cohorts. The mean (±SD) duration of T2D, fasting blood glucose (FBG) level, and Glycosylated hemoglobin (HbA1c) level were 12 (± 5.4) years, 7.9 mmol/L (±3) (142.4 mg/dL), and 7.3 % (± 1.6) (56 mmol/mol), respectively. The CSA of C6 and C7 were significantly smaller (*P* = .03 and 0.042, respectively) in the T2D cohort, while the C5 CSA was similar (*P* = .556) between both cohorts (Table 1).

**Table 1:** Demographic data, laboratory values, and sonographic measurements from the control and type 2 diabetes cohorts.

	Control Cohort (N = 18)		Type 2 Diabetes Cohort (N = 25)		
Gender					
Male N %	11	61.1	16	64	
Female N %	7	38.9	9	36	
	Mean	± SD	Mean	± SD	*P*
Age (years)	51.6	14.6	58.5	10.6	.099
Height (cm)	161.2	14.1	163.9	6.9	.453
Weight (kg)	84.9	15.6	78.5	14.9	.181
Body mass index (kg/m^2^)	31.3	5.6	29.5	4.9	.285
Duration of T2D (years)			12	5.4	
FBG (mmol/L)			7.9	3	
HbA1c (%)			7.3	1.6	
C5 CSA (mm^2^)	4.7	1.5	4.5	1.9	.556
C6 CSA (mm^2^)	5.7	2.9	4.5	2.2	.030*
C7 CSA (mm^2^)	6.2	3.9	4.8	2.6	.042*

CSA = cross-sectional area, FBG = fasting blood glucose, HbA1c = Glycosylated Hemoglobin, T2D = Type 2 diabetes.

* *P* < .05.

In the T2D cohort, HbA1c, FBG, and the duration of T2D were negatively correlated with the C5, C6, and C7 CSAs. Body weight was negatively correlated with the C7 CSA. No other statistically significant correlations were identified between other demographic data from the T2D cohort and the nerve root CSAs (Table 2). Furthermore, no significant correlations were identified between demographic data from the control cohort and the nerve root CSAs (Table 3).

**Table 2:** Correlation between the C5, C6, and C7 cross-sectional areas and the demographic and clinical data of the type 2 diabetes cohort.

		C5 CSA	C6 CSA	C7 CSA
Age	Pearson Correlation	−0.014	0.149	0.187
	Sig. (2-tailed)	0.924	0.308	0.199
	N	49	49	49
Weight in kg	Pearson Correlation	−0.275	−0.136	−0.349*
	Sig. (2-tailed)	0.055	0.352	0.014
	N	49	49	49
Height in cm	Pearson Correlation	−0.193	−0.196	−0.197
	Sig. (2-tailed)	0.184	0.178	0.176
	N	49	49	49
Body mass index	Pearson Correlation	−0.161	−0.024	−0.249
	Sig. (2-tailed)	0.270	0.869	0.084
	N	49	49	49
HbA1c	Pearson Correlation	−0.417**	−0.303*	−0.332*
	Sig. (2-tailed)	0.003	0.034	0.020
	N	49	49	49
FBG	Pearson Correlation	−0.371**	−0.294*	−0.294*
	Sig. (2-tailed)	0.009	0.041	0.041
	N	49	49	49
Duration of T2D	Pearson Correlation	−0.494**	−0.321*	−0.334*
	Sig. (2-tailed)	0.000	0.025	0.019
	N	49	49	49

CSA = cross-sectional area, FBG = fasting blood glucose, HbA1c = Glycosylated Hemoglobin, T2D = Type 2 diabetes.

* Correlation is significant at the 0.05 level (2-tailed).

** Correlation is significant at the 0.01 level (2-tailed).

**Table 3:** Correlation between the C5, C6, and C7 cross-sectional areas and the demographic data of the control cohort.

		C5 CSA	C6 CSA	C7 CSA
Age	Pearson Correlation	−0.262	−0.061	0.093
	Sig. (2-tailed)	0.123	0.724	0.589
	N	36	36	36
Weight in kg	Pearson Correlation	−0.211	−0.051	−0.173
	Sig. (2-tailed)	0.216	0.770	0.313
	N	36	36	36
Height in cm	Pearson Correlation	0.038	−0.020	−0.024
	Sig. (2-tailed)	0.825	0.907	0.890
	N	36	36	36
Body mass index	Pearson Correlation	−0.150	−0.029	−0.109
	Sig. (2-tailed)	0.382	0.868	0.528
	N	36	36	36

CSA = cross-sectional area.

## 4. Discussion

The classification of diabetic polyneuropathy includes focal or multifocal neuropathies including radiculoplexus neuropathies. The radiculoplexus neuropathies can involve the entire brachial plexus, the roots, or the nerve branches and are reported to involve cervical, thoracic or lumbosacral segments.^[2]^

Magnetic resonance imaging is the imaging modality of choice for the brachial plexus and peripheral nerves. However, it is expensive, time consuming, and not readily available in all health care centers. Alternatively, US can be used as a reliable, cost-effective, convenient, accessible, and high-resolution method.^[10,11]^

In the present study we observed smaller CSAs of C6 and C7 in patients with T2D compared to the control cohort. Hypotheses concerning the multiple etiologies of diabetic neuropathy include a metabolic insult to nerve fibers, neurovascular insufficiency, autoimmune damage,

and neurohormonal growth factor deficiency.^[12]^ Current research on diabetic neuropathy is focused on oxidative stress, advanced glycation-end products, protein kinase C, and the polyol pathway.^[13]^ Diabetic polyneuropathy is associated with axonal and neuronal degeneration along with impaired peripheral nerve regeneration The early deficient regeneration will later lead to decreased myelin thickness and axonal diameter.^[14]^ In previous study that studied the CSA of the vagus nerve in diabetic patients showed small CSA in diabetic patients and attributed this vagus nerve atrophy to vagus neuropathy or degeneration that can occur in diabetic patients.^[15]^ This vagus nerve degeneration has been described in patients with diabetes in other studies.^[16,17]^ It has been reported that, patients with diabetic cervical and lumbosacral radiculoplexus neuropathy had evidence of ischemic injury with active axonal degeneration, and multifocal fiber loss, along with microvasculitis which were more striking in the case of DCRPN.^[2]^ These pathological changes might occur early in asymptomatic patients, and axonal degeneration along with fiber loss could be the basis of the smaller CSA in our patients. In the acute or subacute stage of DCRPN, magnetic resonance imaging shows variable abnormalities including abnormally hyperintense T2 signal in the edematous nerve, diffuse nerve enlargement, fascicular enlargement, contrast enhancement, muscle denervation edema, and fatty atrophy of the muscle in the chronic stage.^[2]^ Previous brachial plexus imaging was done during the acute or subacute stages of DCRPN where there is fulminant inflammatory process.^[2]^ However, our study conducted in the preclinical asymptomatic stages at which time the pathological process is not acute or slowly progressive. Katz et al reported that the motor deficits of DCRPN affect mainly the intrinsic hand muscles and the flexor and extensor muscles of the wrist.^[18]^ The intrinsic hand muscles are supplied mainly by the C8 and T1 nerve roots, the flexor and extensor muscles of the wrist are innervated by the C6 and C7 nerve roots, and C5 innervates mainly the shoulder muscles.^[19]^ The prominent involvement of the flexors and extensors of the wrist in DCRPN may support our observation of significantly small CSAs of C6 and C7 in asymptomatic patients with T2D, which could be due to early predilection of the pathological process to the C6 and C7 roots.

We found a negative correlation between the duration of T2D and the FBG and HbA1c levels to the CSA of the examined nerve roots. In a study by Massie et al, patients with DCRPN had a short duration of T2D, and good glycemic control.^[2]^ A similar observation was reported in other studies.^[20,21]^ However, they did not test for the correlation between T2D parameters and the severity of the symptoms. In partial accordance with our results, in asymptomatic patients with T2D, Gregerson reported progressive subclinical nerve conduction abnormalities that were correlated with the duration of T2D.^[22]^ Moreover, a strong correlation between the length of exposure to hyperglycemia and the degree of neuropathy was reported by another study.^[23]^ It has been reported that, good glycemic control can delay and likely prevent the development of diabetic peripheral neuropathies.^[21]^ These changes may reflect subclinical brachial plexus nerve root involvement. If this is confirmed in future studies, then, perhaps, brachial plexus nerve root ultrasound can be used for the early detection and possible preventive plane of DCRPN.

We recognize the limitations of this study. First, we had a small number studied subjects, however, despite this we had statistically significant findings. An additional limitation is the lack of comparison with symptomatic patients. A future study with larger cohorts and with symptomatic cohorts would be expected to be beneficial.

## 5. Conclusion

Our study demonstrated smaller CSAs of the brachial plexus nerve roots in asymptomatic patients with T2D when compared to normal controls. Furthermore, the smaller CSA correlated negatively with the clinical features of T2D.

## Acknowledgments

The authors thank the Deanship of Scientific Research at Prince Sattam Bin Abdulaziz University.

## Author contributions

**Conceptualization:** Mamdouh Ali Kotb, Mohamed A. Bedewi, Daifallah Mohamed Almalki, Ali Abdullah AlAseeri, Kholoud J. Sandougah, Steven B. Soliman, Nasser M. Aldossary, Wael Hamed Aboulela.

**Data curation:** Mamdouh Ali Kotb, Mohamed A. Bedewi.

**Formal analysis:** Mamdouh Ali Kotb, Mohamed A. Bedewi.

**Funding acquisition:** Mamdouh Ali Kotb.

**Investigation:** Mamdouh Ali Kotb, Mohamed A. Bedewi.

**Methodology:** Mamdouh Ali Kotb, Mohamed A. Bedewi, Ali Abdullah AlAseeri.

**Project administration:** Mamdouh Ali Kotb.

**Resources:** Mohamed A. Bedewi, Daifallah Mohamed Almalki, Nasser M. Aldossary.

**Software:** Mamdouh Ali Kotb, Mohamed A. Bedewi.

**Supervision:** Mamdouh Ali Kotb, Kholoud J. Sandougah, Nasser M. Aldossary, Wael Hamed Aboulela.

**Validation:** Mamdouh Ali Kotb.

**Visualization:** Mamdouh Ali Kotb, Mohamed A. Bedewi.

**Writing – original draft:** Mamdouh Ali Kotb.

**Writing – review & editing:** Mamdouh Ali Kotb, Mohamed A. Bedewi, Steven B. Soliman.
